# Dynamic Gesture Recognition Using Surface EMG Signals Based on Multi-Stream Residual Network

**DOI:** 10.3389/fbioe.2021.779353

**Published:** 2021-10-22

**Authors:** Zhiwen Yang, Du Jiang, Ying Sun, Bo Tao, Xiliang Tong, Guozhang Jiang, Manman Xu, Juntong Yun, Ying Liu, Baojia Chen, Jianyi Kong

**Affiliations:** ^1^ Key Laboratory of Metallurgical Equipment and Control Technology of Ministry of Education, Wuhan University of Science and Technology, Wuhan, China; ^2^ Research Center for Biomimetic Robot and Intelligent Measurement and Control, Wuhan University of Science and Technology, Wuhan, China; ^3^ Hubei Key Laboratory of Mechanical Transmission and Manufacturing Engineering, Wuhan University of Science and Technology, Wuhan, China; ^4^ Institute of Precision Manufacturing, Wuhan University of Science and Technology, Wuhan, China; ^5^ Hubei Key Laboratory of Hydroelectric Machinery Design and Maintenance, Three Gorges University, Yichang, China

**Keywords:** dynamic gesture recognition, sEMG, MResLSTM, signal fusion, deep neural network

## Abstract

Gesture recognition technology is widely used in the flexible and precise control of manipulators in the assisted medical field. Our MResLSTM algorithm can effectively perform dynamic gesture recognition. The result of surface EMG signal decoding is applied to the controller, which can improve the fluency of artificial hand control. Much current gesture recognition research using sEMG has focused on static gestures. In addition, the accuracy of recognition depends on the extraction and selection of features. However, Static gesture research cannot meet the requirements of natural human-computer interaction and dexterous control of manipulators. Therefore, a multi-stream residual network (MResLSTM) is proposed for dynamic hand movement recognition. This study aims to improve the accuracy and stability of dynamic gesture recognition. Simultaneously, it can also advance the research on the smooth control of the Manipulator. We combine the residual model and the convolutional short-term memory model into a unified framework. The architecture extracts spatiotemporal features from two aspects: global and deep, and combines feature fusion to retain essential information. The strategy of pointwise group convolution and channel shuffle is used to reduce the number of network calculations. A dataset is constructed containing six dynamic gestures for model training. The experimental results show that on the same recognition model, the gesture recognition effect of fusion of sEMG signal and acceleration signal is better than that of only using sEMG signal. The proposed approach obtains competitive performance on our dataset with the recognition accuracies of 93.52%, achieving state-of-the-art performance with 89.65% precision on the Ninapro DB1 dataset. Our bionic calculation method is applied to the controller, which can realize the continuity of human-computer interaction and the flexibility of manipulator control.

## Introduction

**FIGURE 1:**
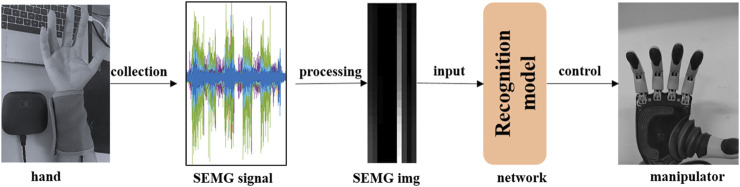
Dexterous hand control process with sEMG signal.

The deep neural network is an intelligent heuristic algorithm used to solve complex real-world problems (He and Jiang, 2020). For example, deep learning is used for data mining to analyze user needs (Chen et al., 2021). The main purpose of the research on dynamic gesture recognition is to promote the development of dynamic human-computer interaction. The dynamic gesture recognition model is applied to the controller of the manipulator, which can improve the continuity and flexibility of the manipulator control. The surface electromyography signal (sEMG) contains a lot of information and can be used for gesture recognition and force prediction (Ma et al., 2020; Atzori et al., 2016; Sadikoglu et al., 2017; Baldacchino et al., 2018). Therefore, it is convenient and feasible to use it as an information interaction medium for human-computer interaction (Sun et al., 2020a; Hu et al., 2019; Jiang et al., 2019a; Shahzad et al., 2019). In biomedical signals, sEMG signals are widely accepted and decoded due to their neural basis and ease of use, so gesture recognition based on sEMG has become a research hotspot in manipulators and human-computer interaction (Xiao et al., 2021; Ahn et al., 2020; Gowtham et al., 2020). Many studies have found that sEMG-based deep learning approaches have great potential in gesture recognition. The gesture recognition model is applied to the controller of the Manipulator to control its actions (Rodríguez-Tapia et al., 2020). The control flow is shown in Figure 1.

Surface sEMG signals represent a promising method to decode the movement intentions of amputees and control multifunctional dexterous hands in a non-invasive manner. The focus of sEMG signal research was to develop pattern recognition and classification techniques for detecting different hand movements. Therefore, many technologies, including fuzzy systems, neural networks, fuzzy support vector machines (SVM), hidden Markov models (HMM), and principal component analysis (PCA), have shown the high accuracy of hand motion recognition (Mendes Junior et al., 2020; Sun et al., 2020b; Cheng et al., 2021; Liao et al., 2021). Secondly, it is mainly from designing better features to improve the accuracy of the gesture recognition network. Then the process of feature extraction and selection is complicated. Third, different feature combinations have other recognition effects on the same model (Duan et al., 2021; Yu et al., 2019; Jiang et al., 2021a). However, deep learning can automatically learn the characteristics of sEMG and avoid the disadvantages of manually extracting the features. Unlike vision-based gesture recognition methods, sEMG-based gesture recognition is not affected by the surrounding environment, such as background lighting and occlusion (Jiang et al., 2019b; Tian et al., 2020; Mujahid et al., 2021). However, different arm positions, electrode displacements, signal non-stationarity, and force changes greatly affect the accuracy and robustness of the sEMG-based recognition model. Finally, only relying on sEMG for gesture recognition cannot fully characterize the features of gestures in motion, making it difficult for the recognition model to converge during training. Therefore, Signal fusion technology is adopted to improve the accuracy and robustness of the network (Xu Zhang et al., 2011; Sun et al., 2018; Tan et al., 2020).

Dynamic gestures are a set of continuous motion gestures to represent a specific meaning, generally including hand movements and arm movements. In the paper, deep learning methods are used to analyze dynamic hand movements. The residual model and variant ConvLSTM model combined into a multi-stream network. For a multi-stream network, each stream independently learns representative features by ResNet. Then, it fuses the features learned from all streams into a unified feature map. Simultaneously, a dual-stream classifier fused with sEMG and ACC signals is used to recognize various dynamic actions to improve the accuracy of behavioral action recognition. The proposed MResLSTM can directly input the preprocessed EMG signal into the network for dynamic gesture recognition. The contributions of this paper are as follows:

1) Surface EMG signals and ACC signals are collected to construct datasets containing six different dynamic gestures.
2) Embedding the SE unit into the Residual module can effectively solve channel dependence. At the same time, the strategy of pointwise group convolution and channel shuffle is adopted to reduce the calculation amount of the model.
3) The proposed MResLSTM achieves state-of-the-art results in terms of dynamic hand movement recognition.

The rest of this paper is organized as follows: *Related Work* discusses the related work, followed by the MResLSTM designed in *Method* and the optimization of the model. *Experiment* shows the experimental results and analysis, and *Conclusion* concludes the paper with a summary and future research directions.

## Related Work

Surface EMG signals is a non-invasive technique for measuring the electrical activity of muscle groups on the skin surface, which makes it a simple and straightforward method that allows the user to actively control the prosthesis (Takaiwa et al., 2011; Gregory and Ren, 2019; Wu et al., 2017). The basic principle of the human-machine interface based on surface EMG signals is to convert sEMG into controllable signals through algorithms such as machine learning. With the precision, portability, and signal processing algorithm performance of the acquisition system, the high reliability of the man-machine interface and the robustness of the prosthetic hand control have become a reality. Recently, many researchers have paid more attention to deep learning in the field of EMG pattern recognition. It can automatically learn features of different abstract levels from many input samples, thereby avoiding cumbersome feature extraction and optimization processes and realizing end-to-end EMG gesture recognition (Weng et el., 2021; Su et al., 2021; Tsinganos et al., 2019; Chaiyaroj et al., 2019).

Atzori et al. (2016) proposed a LeNet-based convolutional neural network model AtzoriNet for end-to-end EMG gesture recognition. He et al. (2018) combined a Long short-term memory network and multilayer perceptrons and conducted experiments on the NinaPro DB1 dataset. When classifying the 52 hand movements of 27 subjects, the accuracy rate reached about 75%. Hu et al. (2019) proposed a CNN model based on the attention mechanism and tested it on the NinaProDB1, NinaProDB2, BioPatRec subdatabase, CapgMyo subdatabase, and csl-hdemg database. Its accuracy rates are 87.0, 82.2, 94.1, 99.7 and 94.5% respectively. Geng et al. (2016) proposed GengNet for gesture recognition based on transient EMG signals. They applied a pre-training strategy to make the EMG gesture recognition performance of the network surpassed the method of extracting signal features and inputting traditional classifier models for gesture recognition. Wu et al. (2018) proposed LSTM-CNN for the dynamic recognition of gestures. Mendes Junior et al., 2020 investigated multiple classification techniques for six hand gestures acquired from 13 participants using eight channels sEMG armband with a sampling rate of 2 kHz. Their best result, with an average accuracy of 94% was obtained from 40 features with the large margin nearest neighbor (LMNN) technique. Côté-Allard et al. (2020) presented an analysis of the features learned using deep learning to classify 11 hand gestures using sEMG. The LSTM model is used to extract timing information in signals. The CNN model can perform secondary feature extraction and signal classification (Peng et al., 2020).

As mentioned above, it is obvious that deep learning methods can overcome the limitation of feature engineering for better feature quality. Many studies have shown that the accuracy of using DNN to classify surface EMG signals is generally higher. However, EMG signal recognition based on deep learning models is expected to improve accuracy and feature extraction complexity (Jiang et al., 2019c; He et al., 2019; Sri-iesaranusorn et al., 2021).

## Method

**FIGURE 2:**
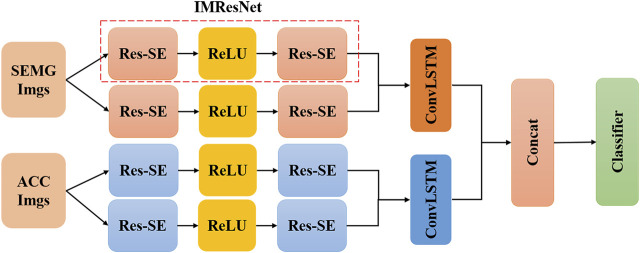
The overall framework of the MResLSTM.

The advantage of dynamic gesture research lies in the ability to apply the trained model to the control of dexterous hands. Dynamic gestures are a set of continuous motion gestures to represent a specific meaning. The dynamic hand movement is regarded as a dynamic transfer action in which one gesture posture is converted to another (Zhang and Li, 2019; Zhang et al., 2021; Liu et al., 2021). In this paper, we formulate the sEMG-based gesture recognition problem as a DNN based image classification problem. In the context of dynamic gesture recognition, the EMG signal has a strong timing. Instantaneous sEMG images and simple classifiers may not fully capture the time information between multiple frames, so a time window is used to sample the sEMG signal, and the sEMG signal is converted to an sEMG image within the time window. In this paper, the MResLSTM is proposed for dynamic gesture recognition, and its overall framework is shown in Figure 2.

The model includes two stages: feature extraction and feature fusion. First, the original sEMG image is decomposed into n patches of equal size. Then these patches are input into a multi-stream network, and each stream independently learns representative features by IMResNet. During the fusion stage, it fuses the features learned from all streams into a unified feature map. The convolutional long short-term memory extracts spatiotemporal feature information from local, global and deep aspects, and combines feature fusion to alleviate the loss of feature information. Finally, the feature map is input to the classifier for classification. To prevent over-fitting, the ReLU nonlinear function is applied after each fully connected layer, batch normalization is performed, and a 50% dropout layer is added after the fully connected layer. Many studies have found that the recognition effect of information fusion technology is better than that of single information. Therefore, this paper proposes a novel dynamic gesture recognition scheme based on the information fusion of sEMG and ACC signals. The original signal is directly converted into images for training the recognition network after preprocessing.

### IMResNet

**FIGURE 3:**
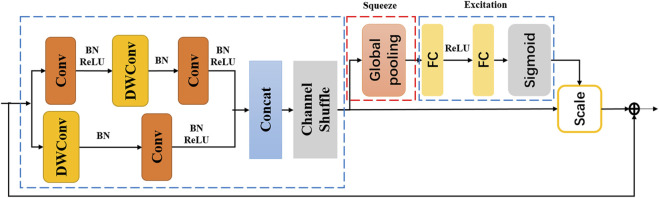
The structure of Res-SE.

The IMResNet module is shown in Figure 2 and consists of two Re-SE units. We embed the SE module into the residual network to form a Re-SE module, the structure of which is shown in Figure 3. The channel relationship of the image constructed by a convolutional neural network through convolution is local. Many researchers hope that the correlation of channels can be explicitly constructed to enhance the feature maps obtained by convolution. Squeeze-Excitation module (SE) is adopted to solve the above issue. This SE module enables the network to increase its sensitivity to signal characteristics to use these feature information in subsequent conversions. The SE module is composed of Squeeze and Excitation, as shown in Figure 3.

The Squeeze compresses the global information to each channel for description through global pooling, effectively solving channel dependence. The output formula of the nth channel after global pooling is as follows:

$$z_{n}=\frac{1}{H\times W}\sum_{i=1}^{H}\sum_{j=1}^{W}I_{n}\left(i,j\right)n=1,2,...,N,$$ (1)

Where *I*_*n*_ is the nth channel of the characteristic image; *H* and *W* are the height and width of the image, respectively; *N* is the number of channels of the picture. Global average pooling can make full use of the correlation of the channel, effectively shield the distribution information in the space, and make the calculation of the output characteristic information more accurate. After squeezing, the Excitation is used to capture the dependence of the channel fully. The Excitation is implemented with 2 fully connected layers. The full connection can use the correlation between channels to train the accurate image scale. The first fully connected layer compresses all channels *C* into *C/k* channels (*k* is the compression ratio). The second fully connected layer is restored to the original *N* channel. The purpose is to reduce the amount of calculation.

The dynamic gesture recognition has real-time requirements, so it is necessary to carry out a lightweight design to reduce network calculation. This paper adopted group convolution and channel shuffle, which greatly reduces the computational complexity of the model while maintaining accuracy. Group convolution minimizes the amount of calculation of the network, but it causes the feature information between different groups to not be exchanged. The core design concept of ShuffleNet is to rearrange different channels to solve the drawbacks caused by grouped convolution (Zhang et al., 2019; Li et al., 2020). The channel reorganization of the feature map after the group convolution ensures that the information can flow between different groups. The IMResNet can directly input the processed EMG image and automatically extract the features of the image.

### Variant ConvLSTM

**FIGURE 4:**
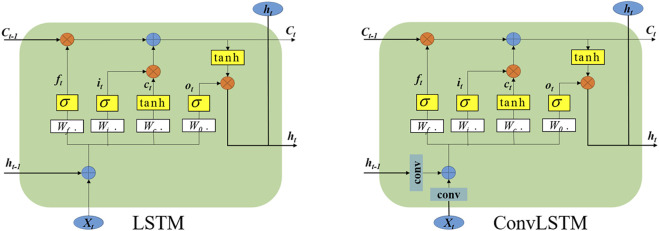
The structure of LSTM and ConvLSTM.

The surface EMG signal of dynamic gestures has a strong timing, so a timing network must be used to extract the timing characteristics of the signal. In this article, we improve the LSTM network structure. The LSTM unit has three thresholds: input gate *i*_*t*_, forget gate *f*_*t,*_ and output gate *o*_*t*_. The subscript t represents the time. In addition, use *c*_*t*_ to represent the cell state of the LSTM at time t. The LSTM network can process time-series data, but if the time series data is an image, adding a convolution operation based on LSTM will be more effective for image feature extraction. The ConvLSTM is a variant of LSTM (Peng et al.,). It not only can extract time-series features but also can describe spatial features. The structure of the LSTM cell and ConvLSTM cell is shown in Figure 4. The main change is that the weight calculation of W has become a convolution operation so that the characteristics of the image can be extracted.

$$\begin{array}{l} f_{t}=\sigma \left(W_{\mathrm{fh}}.h_{t-1}+W_{\mathrm{fx}}.x_{t}+b_{f}\right)\\ i_{t}=\sigma \left(W_{ih}.h_{\mathrm{t-1}}+W_{\mathrm{ix}}.x_{t}+b_{f}\right)\\ C_{t}=f_{t}\circ C_{t-1}+i_{t}\circ \tanh\left(W_{\mathrm{ch}}.h_{\mathrm{t-1}}+W_{\mathrm{cx}}.x_{t}+b_{c}\right]\\ o_{t}=\sigma \left(W_{\mathrm{oh}}.h_{t-1}+W_{\mathrm{ox}}.x_{t}]+b_{o}\right)\\ h_{t}=o_{t}\circ \tanh\left(C_{t}\right). \end{array}$$ (2)

Eq. 2 is the calculation formula of the LSTM unit. Where *x*_*t*_ is the input, *C*_*t*_ is the cell state, *h*_*t*_ is the hidden state. “◦” represents the Hadamard product.

$$\begin{array}{l} f_{t}=\sigma \left(W_{\mathrm{fh}}*H_{t-1}+W_{\mathrm{fx}}*X_{t}+b_{f}\right)\\ i_{t}=\sigma \left(W_{\mathrm{ih}}*H_{t-1}+W_{\mathrm{ix}}*X_{t}+b_{f}\right)\\ C_{t}=f_{t}\circ C_{t-1}+i_{t}\circ \tanh\left(W_{\mathrm{ch}}*H_{t-1}+W_{\mathrm{cx}}*X_{t}+b_{c}\right]\\ o_{t}=\sigma \left(W_{\mathrm{oh}}*h_{t-1}+W_{\mathrm{ox}}*x_{t}]+b_{o}\right)\\ h_{t}=o_{t}\circ \tanh\left(C_{t}\right). \end{array}$$ (3)

**FIGURE 5:**
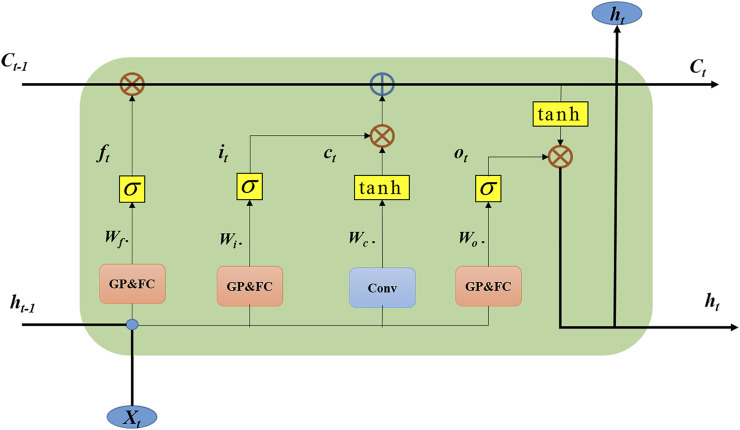
The structure of Variant ConvLSTM.

Eq. 3 is the calculation formula of the ConvLSTM unit. Where *X*_*t*_ is the input, *C*_*t*_ is the cell state, and *H*_*t*_ is the hidden state. “*” represents the convolutional operations, and “◦” means the Hadamard product. The ConvLSTM has a large number of parameters due to the convolution operation. In addition, the convolution in ConvLSTM has no spatial attention effect. The convolution of the three gates hardly affects the Spatio-temporal feature fusion. Therefore, reducing the convolution operation in the three gates can obtain better accuracy, fewer parameters and lower computational cost. This variant of ConvLSTM is improved on the basis of ConvSTLM, as shown in Figure 5.

The Variant ConvLSTM only retains the convolution at the input state in the ConvLSTM structure. The rest of the convolution operations are replaced by global average pooling and fully connected operations. The working principle of VConvLSTM can be expressed by:

$$\begin{array}{l} f_{t}=\sigma \left(W_{\mathrm{fh}}\mathrm{GP}\left(H_{t-1}\right)+W_{\mathrm{fx}}\mathrm{GP}\left(X_{t}\right)+b_{f}\right)\\ i_{t}=\sigma \left(W_{\mathrm{ih}}\mathrm{GP}\left(H_{t-1}\right)+W_{\mathrm{ix}}\mathrm{GP}\left(X_{t}\right)+b_{f}\right)\\ C_{t}=f_{t}\circ C_{t-1}+i_{t}\circ \tanh\left(W_{\mathrm{ch}}*H_{t-1}+W_{\mathrm{cx}}*X_{t}+b_{c}\right]\\ o_{t}=\sigma \left(W_{oh}GP\left(H_{t-1}\right)+W_{ox}GP\left(X_{t}\right)]+b_{o}\right)\\ h_{t}=o_{t}\circ \tanh\left(C_{t}\right). \end{array}$$ (4)

Eq. 4 is the calculation formula of the Variant ConvLSTM unit. Where *X*_*t*_ is the input, *C*_*t*_ is the cell state, and *H*_*t*_ is the hidden state. “*” represents the fully connected operations, and “◦” represents the Hadamard product. *GP* stands for global average pooling.

### Dataset Acquisition

**FIGURE 6:**
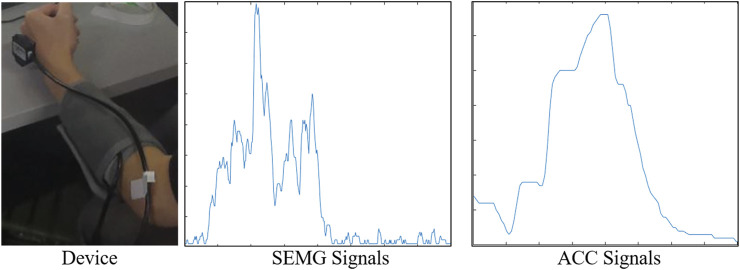
Signal acquisition diagram.

The acquisition of sEMG and acceleration signals is the basis for realizing human hand movement recognition. In this article, a 16-channel SEMG instrument is used for signal acquisition. When the signal is collected, the installation of the equipment is shown in Figure 6.

The ages of the experimenters were distributed among ten persons between 20 and 30 years old. The details of the subjects are summarized in Table 1. The electromyography cuff is worn on the left hand, and the acceleration sensor is close to the back of the hand. During the collection process, the forearm should be kept as level as possible.

**TABLE 1:** Demographic information the subjects.

Subjects	Hand	Status	Age	Sex
S0	Left	Healthy	25	M
S1	Left	Healthy	25	M
S2	Left	Healthy	26	M
S3	Left	Healthy	24	W
S4	Left	Healthy	26	M
S5	Left	Healthy	26	M
S6	Left	Healthy	23	M
S7	Left	Healthy	28	M
S8	Left	Healthy	26	M
S9	Left	Healthy	25	M

**FIGURE 7:**
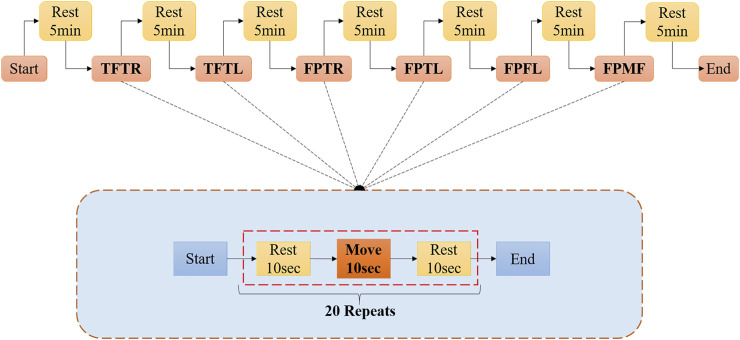
Signal acquisition flowchart.

The sampling frequency is set to 1000 Hz, the motion cycle of different gesture actions is set to 10 s, and a set of experiments are collected 20 times. During the experiment, taking into account the fatigue of the negative muscles, take a five-minute rest after each collection and proceed to the next set of experiments. In each experiment, the repeated method is to rest for 10 s, keep the action for 10 s, repeat twenty times, and collect for three consecutive days, using the same collection method every day. This method can be used to obtain temporal and spatial differences in myoelectric signals of the same individual. The complete paradigm is illustrated in Figure 7.

**FIGURE 8:**
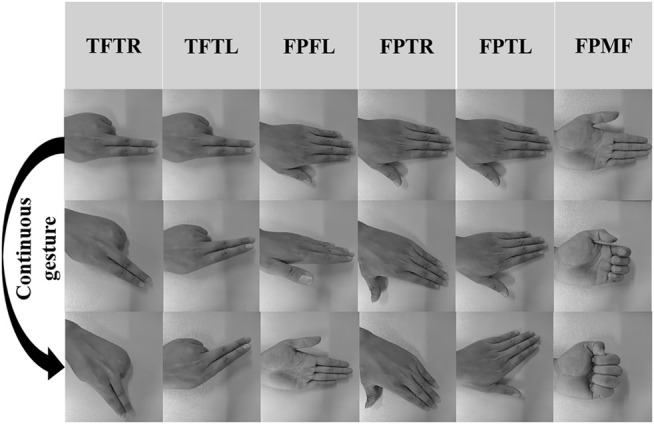
Six dynamic hand movements.

The six gestures involving the entire hand movement are shown in Figure 8, including two-finger left turn (TFTR), two-finger right turn (TFTL), flat palm flip (FPTL), flat palm left turn (FPTL), flat palm right turn (FPTR) and flat palm fist (FPMF).

## Experiment

The dataset is randomly divided into two groups: one is the training set, and the other is the test set. The training set contains 500 sets for each gesture, and each test set contains 60 sets. Experimental environment hardware: Intel(R) Core(TM) i5-10210U CPU@1.60 GHz; memory: 8.00 GB; system type: 64-bit operating system, x64-based processor. All experiments are implemented by PyTorch 1.7.0 + cu110 on NVIDIA GTX 1080Ti GPU.

### Pretreatment

The process of sEMG signals collection is continuous, and the sEMG includes active segment signals and inactive segment information. To improve the accuracy and speed of the recognition model, it is necessary to eliminate non-active segment information. Research shows that the threshold method can efficiently extract active segments. The active segment detection formula is as follows:

$$S\left(n\right)=\sum_{c=1}^{c}{\left(\mathrm{SEM}G_{c}\left(n\right)-\mathrm{SEM}G_{c}^{\mathrm{mean}}\right)}^{2}\geq \mathrm{TH},n=1,2,3,....,n,$$ (5)

Where *c* is the number of acquisition channels of sEMG; *N* is the number of sampling points; *SEMG*_*c*_ *(n)* is the value of the nth sampling point of the c channel; *SEMG*_*c*_^*mean*^ is the average value of the sEMG when the *c* channel is relaxed; *TH* is the set threshold. In this article, *TH* is 15% of the peak energy of each channel.

The raw SEMG contains a lot of noise, and the signal needs to be filtered and noise-reduced. The frequency of the power frequency noise in the environment is mainly concentrated at 50 Hz or the corresponding integer multiple of the frequency. A 20-order comb filter is used to filter it. Wavelet transform can highlight the signal characteristics in the time domain and frequency domain. Wavelet transform is to shift the basic wavelet function and then perform inner product with the signal that needs noise reduction at different scales. The wavelet transform is to shift the basic wavelet function, and then at different scales, the inner product with the signal that needs noise reduction, namely:

$$WT_{x}\left(\alpha ,\tau \right)=\frac{1}{\sqrt{\alpha }}\int_{-\infty }^{+\infty }x\left(t\right)\varphi^{*}\left(\frac{t-\tau }{\alpha }\right)\mathrm{dt},$$ (6)

**FIGURE 9:**
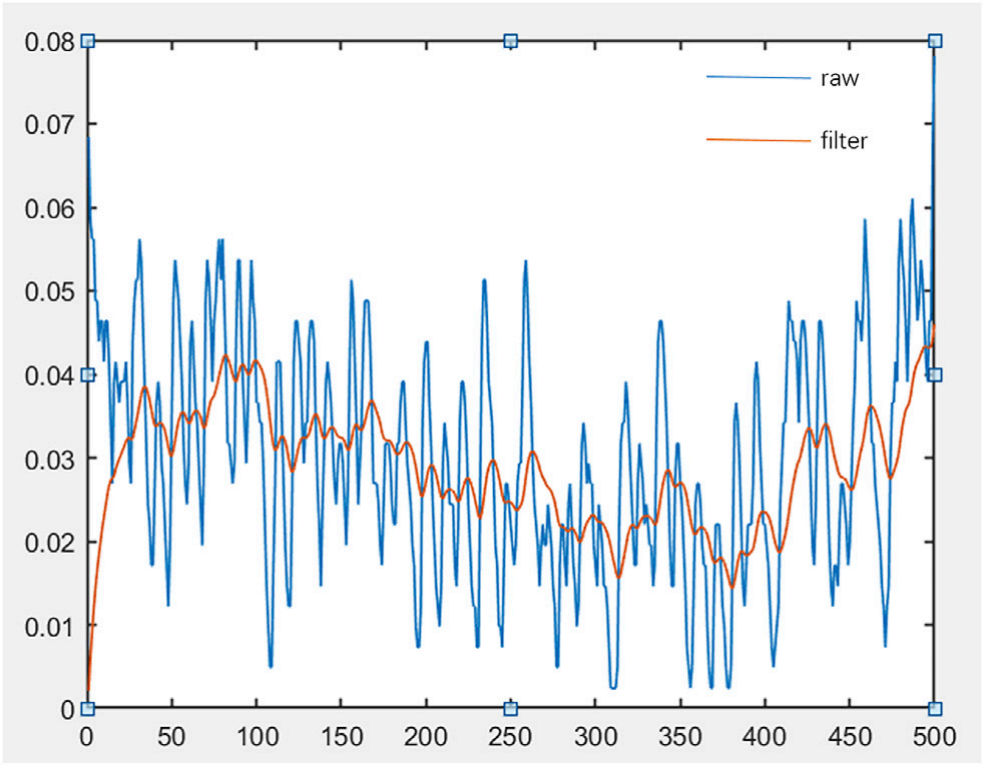
The timing diagram of sEMG.

Where α >0, is the scale factor, and its function is to expand and contract the basic wavelet φ(t) function and τ represents the displacement. In this paper, coif5 is used as the wavelet basis function, and the unbiased likelihood estimation threshold is used for threshold processing and the hard threshold function to process noise signals. The effect after sEMG treatment is shown in Figure 9.

**FIGURE 10:**
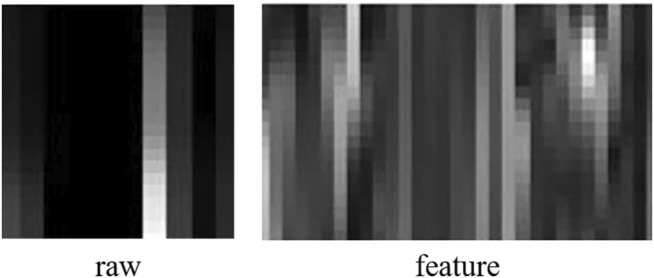
Raw image and feature image.

The proposed recognition network compares the recognition effect of the original EMG image and the multi-EMG feature image as the input source. The raw image and feature image are shown in Figure 10. The dynamic recursive feature selection algorithm is used to calculate the correlation between each EMG feature and the target using mutual information. The EMG feature that is least relevant to the target is eliminated, and the optimal feature is selected.

This paper selects four characteristics: average absolute value (MAV), signal high and low-frequency ratio (FR), median frequency (MDF), and power spectrum average power (MNP) to construct a featured image. The calculation formulas for the four characteristics are as follows:

$$\begin{array}{l} \begin{array}{cc} \mathrm{MAV}=\frac{1}{K}\sum_{i=1}^{K}\left|x_{i}\right| & \mathrm{FR}=\sum_{i=\mathrm{LLC}}^{\mathrm{LHC}}P_{i}/\sum_{i=\mathrm{HLC}}^{\mathrm{HHC}}P_{i} \end{array},\\ \sum_{i=1}^{\mathrm{MDF}}P_{i}=\sum_{i=\mathrm{MDF}}^{M}P_{i}=\frac{1}{2}\sum_{i=1}^{M}P_{i}\mathrm{MNP}=\frac{1}{M}\sum_{i=1}^{M}P_{i}, \end{array}$$ (7)

Where *x*_*i*_ represents the peak value of the i-th point of SEMG in the time sequence; *K* represents the number of signal sampling points. *P*_*i*_ represents the power value of the i-th point of SEMG on the spectrum; *M* is the signal bandwidth. *LLC* and *LHC* are the lower and upper cut-off frequencies of the low-frequency band, respectively; HLC and HHC are the lower and upper cut-off frequencies of the high-frequency band, respectively.

### Experimental Results and Analysis

The calculation amount of a multi-stream network is larger than that of a single network. Therefore, it is necessary to construct a comparative experiment between a multi-stream network and a single network. In the comparison experiment, the input of both recognition models is all the original EMG images. At the same time, no ACC information fusion is added. In addition, the input matrix format of a single network model is different, and the input data format needs to be fine-tuned.

The experimental results are shown in Table 2. It can be seen from Table 2 that the gesture recognition effect of the multi-stream network is better than that of the single network. The multi-stream network can extract more key features and prevent the gradient from disappearing.

**TABLE 2:** results of different networks.

Network model	Accuracy (%)
Single-stream network	73.21
Multi-stream network	84.35

Information fusion increases the workload of data collection, improves the complexity of the network, and reduces the identification efficiency of the network. Therefore, to verify the effectiveness of the fusion acceleration signal, a corresponding comparative experiment was carried out. In the experiments, the acceleration (ACC) signal is input into the network as an independent branch, the raw sEMG image is the input source of the network, and other conditions remain unchanged.

The comparison results are shown in Table 3. Only using SEMG for dynamic gesture recognition, its recognition effect is not as good as information fusion on the same model. The characteristic signals of a variety of signals are not entirely the same. Combining them may produce complementary information. These complementary features can improve the recognition accuracy of the network. However, sometimes information fusion can also lead to information redundancy.

**TABLE 3:** results of information fusion.

Information fusion	Accuracy (%)
ACC	90.71
----	84.35

To judge the effectiveness of feature extraction, the feature image and the original EMG image are used as the input source of the network to conduct a comparative experiment. During the experiment, both networks added ACC signals. The difference is the input source of the network.

The experimental results are shown in Table 4. The recognition effect of the input feature image is better than the original EMG image. The featured image effectively retains the critical information, which significantly improves the recognition accuracy of the multi-stream network.

**TABLE 4:** results of the different input sources.

Input source	Accuracy (%)
Raw img	90.71
Feature img	93.52

**FIGURE 11:**
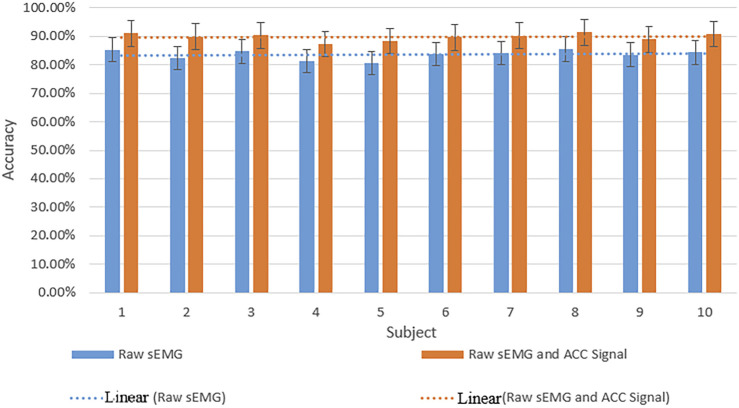
The recognition rate of 10 subjects on MResLSTM.

The average recognition rate of the proposed MResLSTM is 93.52%. However, it can be seen from Figure 11 that the recognition effect of the model is affected by individual differences. Experimental results show that the recognition rate difference between subjects is about 8%. The reason may be that the position of the acquisition instrument has changed or that the hand movement is fast or slow during the signal acquisition process.

To show the advantages of our model, more comparisons with other neural networks should be added, so it is necessary to conduct an experiment on the public dataset Ninapro DB1. The NinaPro DB1 dataset contains 52 different gestures of 27 healthy subjects, different from the gestures contained in the data set used in this article. It is necessary to fine-tune the model’s classifier to enable it to perform 52 classifications. The experimental results are shown in Table 5. Experimental results show that our proposed multi-stream network is better than other algorithms.

**TABLE 5:** Comparison results of different approaches on NinaPro DB1.

Algorithms	Accuracy (%)
Atzori_Net^[Atzori et al.2016]^	66.73
Geng_Net^[Geng et al.2016]^	77.80
Gene_ELM^[Cene et al.2019]^	75.11
Yu_CNN^[Yu et al.2019]^	79.50
GoogleNet	81.27
Wei_MSCNN^[Wei et al.2019]^	85.00
MResLSTM(our)	89.31

Through the comparison of various recognition algorithms in Table 5, it can be seen that the recognition rate of the MResLSTM on the public dataset is 89.31%, which is 4 percentage points higher than MSCNN. It is not difficult to see from the comparative experimental results that with the further development of deep learning in EMG gesture recognition in recent years, the advantages of deep convolutional neural networks in the research of EMG pattern recognition have become more and more apparent. Among them, the average gesture recognition rate based on multi-stream CNN proposed by Wei reached 85.00%. The network is divided into a multi-stream decomposition stage and a fusion stage. In the multi-stream decomposition stage, each stream independently learns representative features through CNN. Then in the fusion stage, it merges the features learned from all streams into a unified feature map and then inputs it into the fusion network to recognize gestures. The experimental results show that the multi-stream network can make up for the single input data information and retain richer features.

**FIGURE 12:**
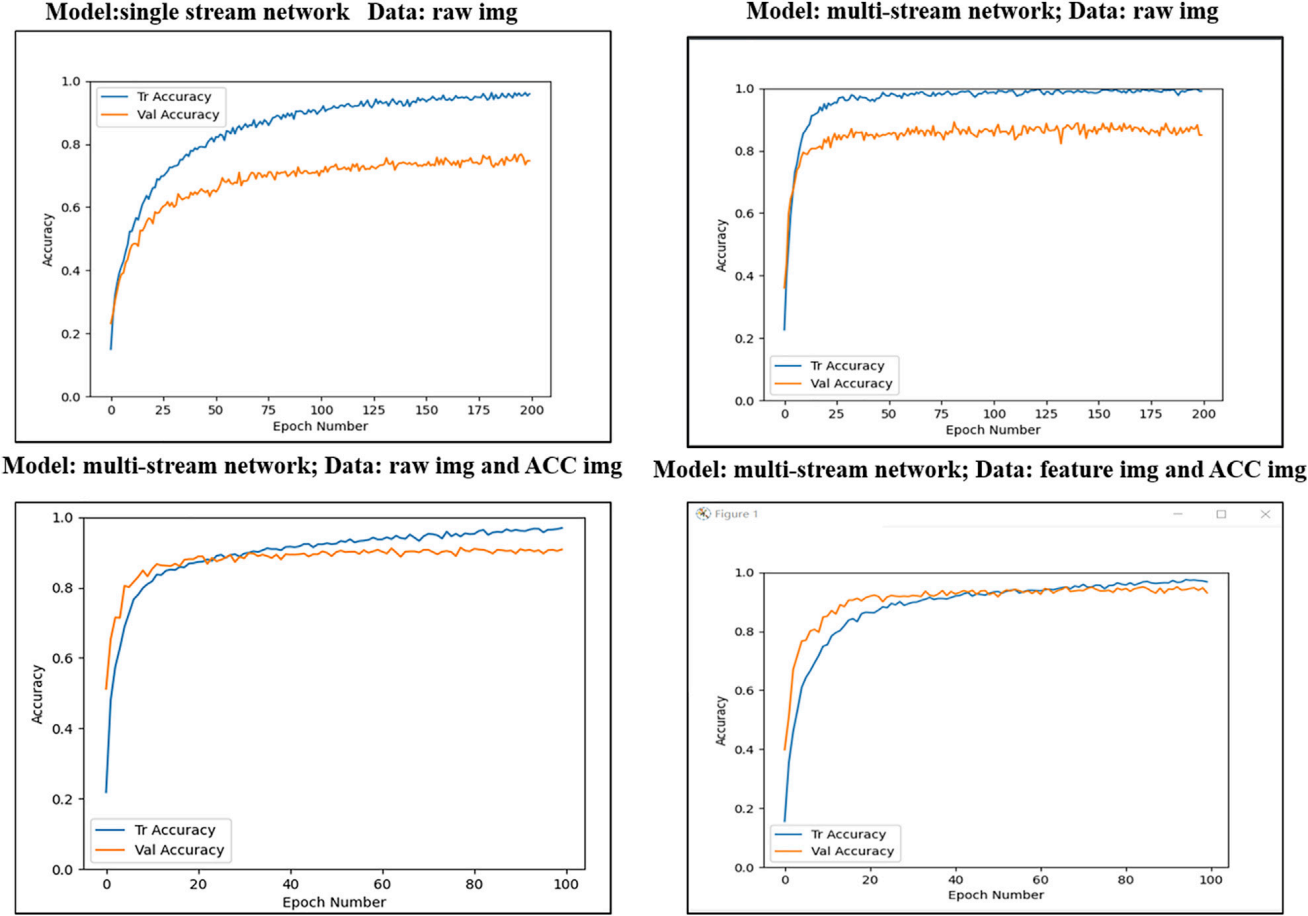
Gesture recognition rate under different conditions.

When the following four experiments are performed, the data batch size is 128, and Epoch is 200. The four experiments are as follows: Experiment 1: the recognition model is a single network structure, and the input source is the original EMG image. Experiment 2: the recognition model is a multi-stream network structure, and the input source is the raw EMG image. Experiment 3: The recognition model is a multi-stream network structure, and the input source is the original EMG and ACC signal image. Experiment 4: The recognition model is a multi-stream network structure, and the input sources are characteristic EMG images and ACC signal images. The training accuracy and verification accuracy during network training are shown in Figure 12.

Comparing Experiment 1 and Experiment 2, it can be seen that the multi-stream network converges faster during training, and the network is more robust. Secondly, the multi-stream network can retain more key features and improve the recognition accuracy of the network. Comparing Experiment 2 and Experiment 3, we can find that Signals fusion can effectively compensate for the shortcomings of single information, making the learned features richer. Comparing Experiment 3 and Experiment 4, we can see that the overall recognition rate of the original EMG image as the input of the network model is low. This is because only limited abstract features can be extracted from the original EMG image through convolution operation.

**FIGURE 13:**
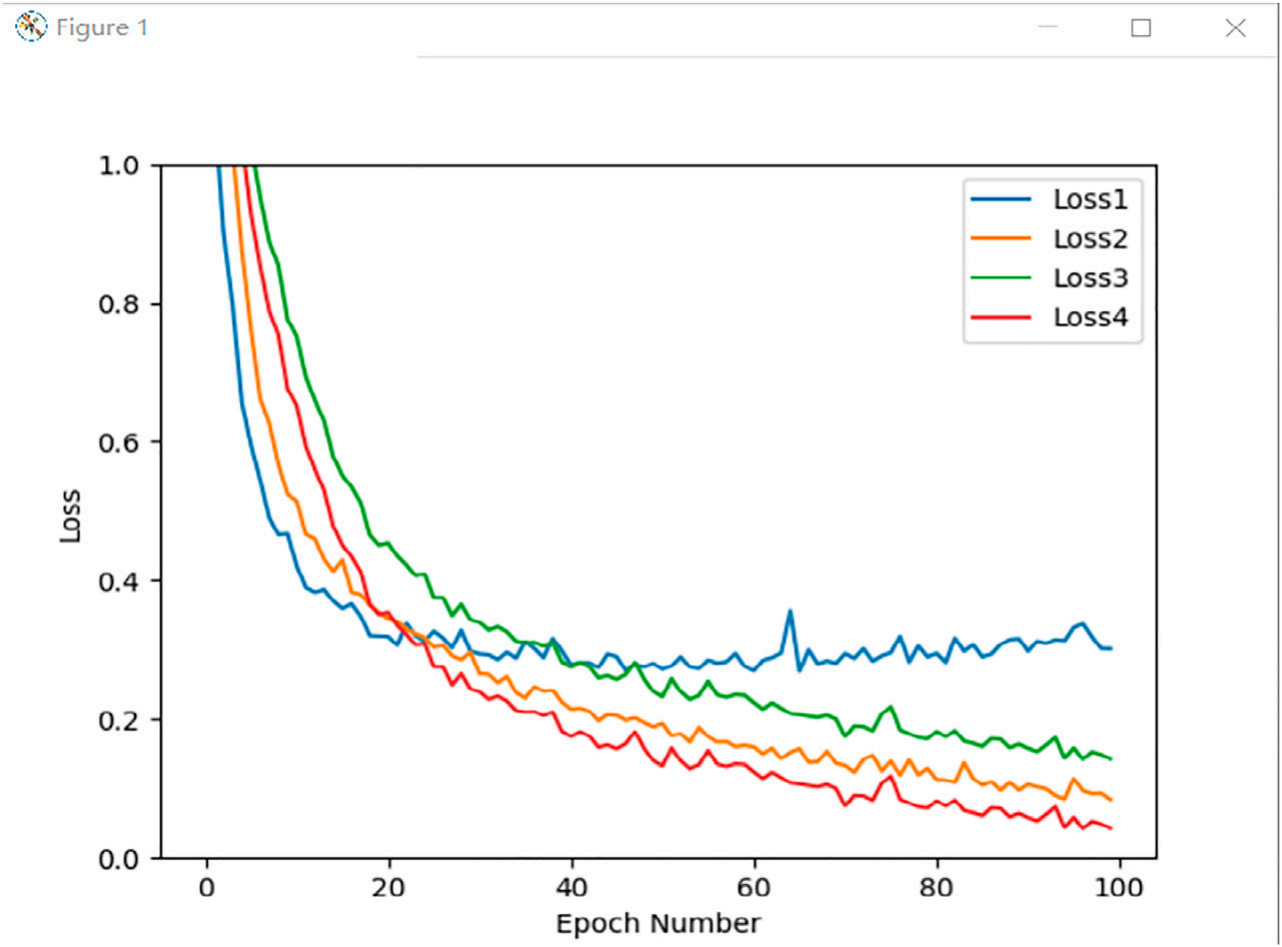
Training loss value under different conditions.

Figure 13 is the training loss graph of four different experiments. Loss1 represents the loss function of Experiment 1, and Loss2 indicates the loss function of Experiment 2. Loss3 means the training loss of Experiment 3, and Loss4 represents the data input is the loss of Experiment 4.

It is not difficult to see from Figure 13 that the network is challenging to converge when a single network is trained with the original sEMG as the input source. This is because a single network has limited features extracted from the sEMG, which is prone to overfitting. The multi-stream network can retain more effective information, making the accuracy and stability of gesture recognition better. Multi-stream networks have better generalization capabilities.

## Conclusion

The motivation of research on dynamic gesture recognition based on sEMG signals is that it can promote the flexible control of manipulators. In this paper, the MResLSTM is proposed for dynamic gestures recognition. The problem of gesture recognition research based on EMG signal is that the amount of data is relatively small and easy to overfit. A multi-stream network structure can retain more crucial information to solve the issue. The strategy of pointwise convolution and channel shuffle is adopted to achieve the real-time requirements of the recognition model. This article uses feature correlation to select key features. The recognition rate of the MResLSTM on the feature image is 93.52%, and the accuracy on the original EMG image is 90.71%. Experimental results show that decent feature images can improve the recognition accuracy of the network. The comparative experiment results on the dataset Ninapro DB1 show our proposed model outperforms the state-of-the-art methods.

SEMG signals are one of the most widely used biological signals to predict the movement intention of the upper limbs. Converting sEMG signals into effective control signals often requires a lot of computing power and complicated processes. The high variability of sEMG and the lack of existing data limit the application of gesture recognition technology (Li et al., 2021; Aranceta-Garza and Conway, 2019). In future work, high-density sEMG (Chen et al., 2020) and multiple information fusion will be the direction of dynamic gesture recognition research. Secondly, the influence of the speed and cycle of hand actions on the model will be a meaningful direction.

## Data Availability

The raw data supporting the conclusions of this article will be made available by the authors, without undue reservation.
